# Neurological Disorders in a Murine Model of Chronic Renal Failure

**DOI:** 10.3390/toxins6010180

**Published:** 2014-01-03

**Authors:** Jean-Marc Chillon, François Brazier, Philippe Bouquet, Ziad A. Massy

**Affiliations:** 1 INSERM U1088, UFR de Pharmacie, 1 rue des Louvels, F-80037 Amiens cedex 1, France; E-Mails: francois.brazier@hotmail.fr (F.B.); philippe.bouquet@u-picardie.fr (P.B.); massy@u-picardie.fr (Z.A.M.); 2 Université de Picardie Jules Verne, Amiens 80025, France; 3 Service de Néphrologie, Centre Hospitalier Universitaire Régional d’Amiens, Amiens 80054, France; 4 Université Paris-Ile-de-France Ouest, Paris, Versailles 78000, France; 5 Service de Néphrologie, Hôpital Ambroise Paré, Paris, Boulogne Billancourt 92100, France

**Keywords:** ischemic stroke, chronic renal failure, recognition, anxiety

## Abstract

Cardiovascular disease is highly prevalent in patients with chronic renal failure (CRF). However, data on the impact of CRF on the cerebral circulatory system are scarce—despite the fact that stroke is the third most common cause of cardiovascular death in people with CRF. In the present study, we examined the impact of CRF on behavior (anxiety), recognition and ischemic stroke severity in a well-defined murine model of CRF. We did not observe any significant increases between CRF mice and non-CRF mice in terms of anxiety. In contrast, CRF mice showed lower levels of anxiety in some tests. Recognition was not impaired (*vs*. controls) after 6 weeks of CRF but was impaired after 10 weeks of CRF. Chronic renal failure enhances the severity of ischemic stroke, as evaluated by the infarct volume size in CRF mice after 34 weeks of CRF. Furthermore, neurological test results in non-CRF mice tended to improve in the days following ischemic stroke, whereas the results in CRF mice tended to worsen. In conclusion, we showed that a murine model of CRF is suitable for evaluating uremic toxicity and the associated neurological disorders. Our data confirm the role of uremic toxicity in the genesis of neurological abnormalities (other than anxiety).

## 1. Introduction

Cardiovascular disease is highly prevalent in patients with chronic renal failure (CRF) and may account for 50% of all deaths in this population [1]. It has been clearly demonstrated that the increased vascular stiffness induced by vascular calcification is associated with cardiovascular alterations (such as left ventricular hypertrophy and a decrease in cardiac stroke volume [2]). In contrast, data on the impact of CRF on the cerebral circulatory system are scarce—despite the fact that stroke is the third most common cause of cardiovascular death in people with CRF. Patients with end-stage renal disease (ESRD) are exposed to a 4- to 10-fold greater risk of hospitalized ischemic and hemorrhagic stroke [3], an increased risk of cognitive impairment and dementia [4,5] and a poor long-term post-stroke prognosis [6] compared with non-ESRD individuals. Furthermore, the prevalence of asymptomatic, silent, brain infarctions is 4 to 5 times higher in dialysis patients than in age- and gender-matched controls [7]. Moreover, patients on dialysis with cognitive impairment appear to have a high number of cortical defects, which are reminiscent of multiple infarct-related damage [8].

The higher frequency of stroke and cognitive impairment in ESRD patients cannot be solely explained by the higher prevalence of traditional [9] and non-traditional risk factors in this population [10]. Other factors (such as the uremic toxins that accumulate during CRF) may account for the altered brain perfusion observed during CRF. Another factor that may be involved in the higher frequency of stroke in patients with CRF is the alteration in endothelial function observed during CRF [11,12,13]. In a murine model of CRF, we recently reported that endothelium-dependent relaxation was impaired in cerebral arterioles (despite the absence of any structural alterations) [14]. This may be related (at least in part) to a decrease in NO levels following an increase in the plasma concentration of asymmetric dimethylarginine [ADMA, a uremic toxin and an endogenous inhibitor of endothelial nitric oxide synthase (eNOS)] and an increase in the quantitative expression of threonine-495-phosphorylated eNOS (the inactive form of eNOS) [14].

Using a well-defined murine model [15], the present study sought to (i) evaluate the impact of CRF (*vs*. control conditions) on anxiety, and exploratory behavior and recognition and (ii) establish whether CRF was associated with an increase in the severity of ischemic stroke.

## 2. Results

### 2.1. Anxiety and Exploratory Behavior

The non-CRF and CRF mice did not differ significantly in terms of bodyweight before CRF induction or 4 or 10 weeks afterwards (results not shown).

#### 2.1.1. The Openfield Test

There were no significant differences between CRF and non-CRF mice in terms of the time spent and distance covered in the corridor or in the center, respectively (results not shown). 

#### 2.1.2. The Dark/Light Box Test

**Figure 1:**
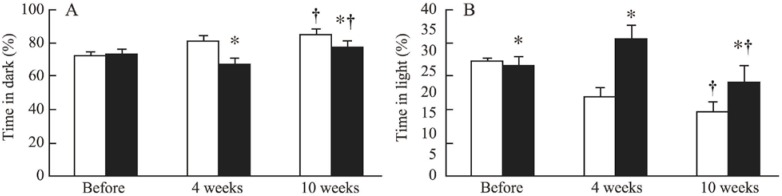
Time spent in the dark (**A**) and time spent in the light (**B**) in non-chronic renal failure (CRF) mice (white bars) and CRF mice (black bars) before CRF induction (before) and after 4 and 10 weeks of CRF. *: *p* < 0.05 CRF *versus* non-CRF; ^†^: *p* < 0.05 10 weeks *versus* before CRF induction.

Compared with non-CRF mice, mice exposed to 4 and 10 weeks of CRF spent significantly less time in the dark (Figure 1).

#### 2.1.3. The Elevated Maze

**Figure 2:**
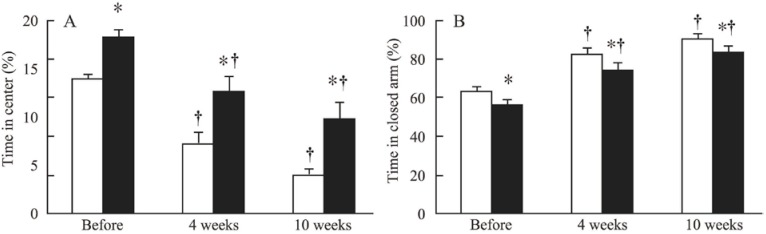
Time spent at the center (**A**) and time spent in the closed arm (**B**) in non-CRF mice (white bars) and CRF mice (black bars) before CRF induction (before) and after 4 and 10 weeks of CRF. *: *p* < 0.05 for CRF *versus* non-CRF; ^†^: *p* < 0.05 for 4 weeks or 10 weeks of CRF *versus* before CRF induction.

Compared with non-CRF mice, mice exposed to 4 and 10 weeks of CRF spent significantly less time in the closed arm and significantly more time in the center (Figure 2). However, mice assigned to the CRF group also spent longer time at the center in the elevated maze (relative to the non-CRF group) before the induction of CRF.

### 2.2. Recognition

Body weight was significantly lower in CRF mice than in non-CRF mice after 6 weeks of CRF (21.1 ± 0.3 g (*n* = 7) *vs.* 22.3 ± 0.3 g (*n* = 9), respectively) and 10 weeks of CRF (21.1 ± 0.3 g (*n* = 7) and 22.3 ± 0.3 g (*n* = 9), respectively (*p* < 0.001).

#### The Y Maze

**Figure 3:**
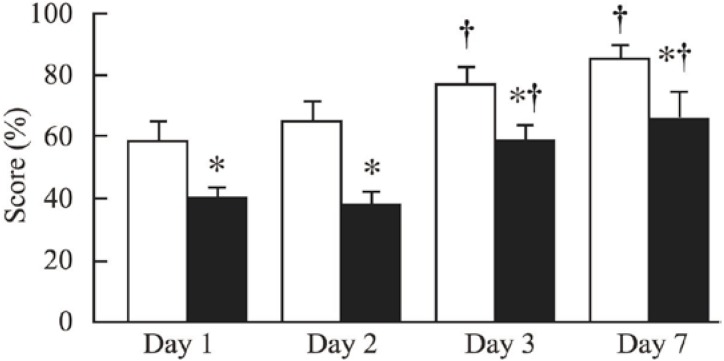
Y maze scores in non-CRF mice (white bars) and CRF mice (black bars) after 10 weeks of CRF. Recognition was tested on days 1, 2, 3 and 7. *: *p* < 0.05 for CRF *versus* non-CRF mice; ^†^: *p* < 0.05 for Day 3 or Day 7 *versus* Day 1.

The CRF-6w and non-CRF-6w mice did not differ significantly in terms of the recognition score (results not shown). In contrast, the recognition score was significantly lower in CRF-10w mice than in non-CRF-10w mice (Figure 3).

### 2.3. Ischemic Stroke

#### 2.3.1. Spontaneous Mortality and Bodyweight

Spontaneous mortality after ischemic stroke was 0% in non-CRF-34w mice and 56% in CRF-34w mice (*p* > 0.05). Three of the CRF-34w mice died between days 0 and 1 and two died between days 2 and 3 (Table 1). Bodyweight was similar in CRF-34w mice and non-CRF-34w mice (Table 1). After ischemic stroke, the bodyweight decreased in both non-CRF-34w and CRF-34w mice (Table 1).

**Table 1:** Number of mice and bodyweight (mean ± SEM) from day 0 (*i.e.*, the day of the ischemic stroke) to day 3 post-stroke in non-CRF-34w and CRF-34w mice. ^†^: *p* < 0.05 for day 1, 2 or 3 *vs.* day 0.

Parameters	non-CRF-34w mice				CRF-34w mice			
Days	Day 0	Day 1	Day 2	Day 3	Day 0	Day 1	Day 2	Day 3
*n*	4	4	4	4	9	6	6	4
Weight (g)	24.8 ± 0.7	24.4 ± 0.8 ^†^	20.8 ± 1.1 ^†^	19.4 ± 0.8 ^†^	24.2 ± 0.4	22.0 ± 0.7 ^†^	19.9 ± 0.8 ^†^	18.7 ± 0.7 ^†^

#### 2.3.2. Neurological Evaluation

Over post-stroke days 1 to 3, the CRF-34w and non-CRF-34w mice did not differ significantly in terms of the neuroscore, the time spent on the rotarod and the prehensile score. However, the neuroscore significantly decreased in CRF-34w mice from day 1 to day 3 but increased in non-CRF-34w mice over the same period. The prehensile score was significantly higher on day 3 than on day 1 in both CRF-34w and non-CRF-34w groups (results not shown).

#### 2.3.3. Histological Evaluation

**Figure 4:**
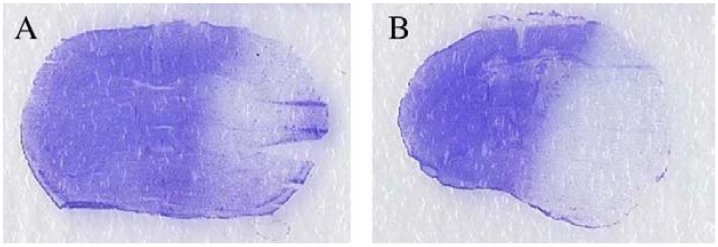
Coronal brain sections (stained with cresyl violet) from 44-week-old mice without CRF (**A**) and after 34 weeks of CRF (**B**).

The total infarct volume was significantly greater in CRF-34w mice than in non-CRF-34w mice (16.8% ± 8.8% *vs.* 46.8% ± 4.9% respectively) (Figure 4).

## 3. Discussion

The present study generated several novel findings. Firstly, we did not observe an increase in anxiety in our murine model of CRF; on the contrary, CRF mice were less anxious in the dark/light test. Secondly, recognition is impaired after 10 weeks of CRF. Thirdly, CRF enhances the severity of ischemic stroke in mice, as assessed by the infarct size after 34 weeks of CRF. Fourthly, the neurological test results in non-CRF mice tended to improve from day 1 to day 3 after ischemic stroke, whereas the results in CRF mice tended to worsen.

### 3.1. Characteristics of the Model

In the present study, we experimented on a well-defined murine model of CRF (characterized notably by a blood urea level of more than 20 mmol/L, relative to below 10 mmol/L in normal mice). We have previously reported that this model is characterized by elevated serum levels of several uremic toxins (including indoxyl sulfate and hippuric acid) and amyloid factors, relative to non-CRF mice. In contrast, levels of uric acid, indole acetic acid, and 3-carboxy-4-methyl-5-propyl-2-furanpropionic acid were similar in CRF and control mice [16]. Most recently, we reported that this model is also associated with endothelial dysfunction, elevated levels of the uremic toxin ADMA (which inhibits NOS), and low levels of L-arginine (the precursor of NO) [14].

### 3.2. Anxiety and Exploratory Behavior

One of our study’s main findings is that anxiety was no higher in CRF mice than in non-CRF mice; on the contrary, CRF mice had lower anxiety levels when assessed in the dark/light box. This finding contradicts with several clinical studies in which anxiety levels are high in both predialysis [17] and hemodialysis CRF patients [17,18]. This apparent discrepancy may be linked to the fact that the results in patients with CRF are related to factors such as low self-esteem, feelings of uselessness and disturbance of the body image. These psychosocial factors may not be relevant in mice. In future work, it would be interesting to examine depression in our murine model of CRF. In fact, depression is linked to the disturbance of neurotransmitter systems. Furthermore, an association between levels of uremic toxins and depression in hemodialysis patients has already been reported [19]. 

In the present study, CRF mice did not show abnormally low levels of locomotor activity or exploratory behavior. This finding agrees with a previous study (in the same murine model) in which locomotor activity in the dark was normal [20]; this was considered to indicate that uremic toxins do not impair the central circadian pacemaker [20].

### 3.3. Recognition

Another important finding in the present study is that recognition (as assessed by the Y maze score) is altered in mice exposed to 10 weeks of CRF. In contrast, this alteration was not present after 6 weeks of CRF. Furthermore, the time spent in the center of the elevated maze (corresponding to the time needed to make a decision) was higher in CRF mice than in non-CRF mice; this may also reflect an alteration in cognitive function. One must interpret these results with caution, since the mice assigned to the CRF group also spent longer time at the center in the elevated maze (relative to the non-CRF group) prior to the induction of CRF. Cognitive impairment has been reported in patients on dialysis and appears to be related to a high numbers of cortical defects, which are reminiscent of multiple infarct-related damage [8]. Other mechanisms (including direct neuronal injury by uremic toxins) may also be involved [21]. In the present study, we did not perform a histological assessment of cortical defects.

### 3.4. The Severity of Ischemic Stroke

One of our study’s most important findings is that infarct cerebral volume was higher in CRF mice than in non-CRF mice. Our results after 34 weeks of CRF are in agreement with clinical reports of a stroke-induced increase in morbimortality in CRF patients [22]. Factors that may contribute to poor post-stroke outcomes in CRF patients might include uremic toxins [23], oxidative stress [24], inflammation [25] and endothelial dysfunction [26]. In a previous study in our murine model, we found that endothelial dysfunction in the cerebral arterioles was associated with an increase in the eNOS inhibitor ADMA [14]. Further investigation of the mechanisms responsible for the increased brain infarct volume in CRF mice is thus required.

Another interesting finding is that neurological test results tended to improve in non-CRF mice and to worsen in CRF mice. This is also in agreement with clinical data from CRF patients [6]. Impaired performance in neurological tests in CRF mice may be due to a lack of recovery of the ischemic penumbra—the part of the brain in which neurons suffer during ischemia but may recover upon reperfusion. Factors possibly associated with post-stroke worsening of the ischemic penumbra again include oxidative stress and inflammation [27,28]. However, one must interpret these data with caution because we observed post-stroke mortality in the CRF mice; this may have artificially improved the neurological test results in the CRF mice by eliminating the most severely affected individuals. Furthermore, factors other than post-stroke worsening of the ischemic penumbra may have contributed to the decrease in neurological test performance. Firstly, the increase in infarct volume in CRF mice may have altered the animals’ motor and sensory abilities and could have resulted in undernutrition and dehydration. Secondly, one of the characteristics of our ischemic stroke model is the ligature of the external carotid. Ligature of the external carotid makes it more difficult for the animal to feed [29]; we did not measure food and fluid intakes in the present experiments and thus where unable to test this hypothesis. 

### 3.5. Study Limitations

Our study had several limitations. Firstly, we did not perform recognition tests before the induction of CRF (*i.e.*, before the electrocoagulation at 8 weeks of age). However, the food restriction implemented before Y maze experiments would probably have increased the perioperative mortality rate.

Secondly, the tests used to examine anxiety and recognition in the present study are used by many other laboratories worldwide. However, other tests of anxiety and recognition might yield different results.

Thirdly, we did not perform neurological tests before stroke induction. In most studies on ischemic stroke, neurological tests are performed both before and after stroke induction so that the mice with the worst pre-stroke scores are not included in the experiments. 

Fourthly, the animals assigned to the CRF group differed significantly from the animals assigned to the non-CRF group in terms of the time spent respectively in the closed arm and in the center of the elevated maze prior to induction of CRF; this interferes with interpretation of the results. Great care was taken to ensure that all mice were exposed to the same experimental conditions. However, a number of extrinsic factors (such as small differences in noise levels) and/or intrinsic factors (such as differences between individual mice) may have influenced the results. For practical reasons, we did not have time to analyze the pre-CRF results before the induction of CRF. This analysis would have enabled us to randomize the mice and ensure that the various groups had similar pre-CRF results.

Fifthly, the mortality rate in the CRF mice was high after induction of ischemic stroke. As mentioned above, this may have biased the results by decreasing the number of mice evaluated in the neurological tests.

Sixthly, we did not measure parameters such as blood pressure, blood glucose levels and body temperature in the present experiments. We have reported previously that our murine model of CRF is not associated with elevated blood pressure [14]. During ischemic stroke induction, mice were kept on a heating pad and then in a warm environment until they had fully recovered from the anesthesia. However, we cannot rule out the possibility that parameters other than uremic toxins were involved in the greater infarct size in CRF mice. Lastly, we did not ascertain whether uremic toxins were specifically involved in the observed alterations. As mentioned above, levels of several uremic toxins are known to be elevated in our murine model of CRF [16]. Further experiments are necessary to fully explore the role of uremic toxins and the underlying disease mechanisms.

## 4. Methods

### 4.1. Animals and Diet

All experiments were performed on female C57BL/6J mice purchased from Charles River Laboratories (Lyon, France). The animals were housed in polycarbonate cages in temperature- and humidity-controlled rooms with a 12-hour/12-hour light/dark cycle and were given standard rations (Harlan Teklad Global Diet 2018, Harlan, Bicester, UK) and tap water *ad libitum*. Animals were handled in accordance with the French legislation. The protocols were approved by an institutional animal care committee (*Comité Régional d’Ethique en Matière d’Experimentation Animale de Picardie*, Amiens, France). 

### 4.2. Induction of CRF

The C57BL/6J mice were randomly assigned to CRF or non-CRF induction groups. To induce CRF, we applied cortical electrocautery to the right kidney at 8 weeks of age and then performed left total nephrectomy at 10 weeks of age in mice anesthetized with ketamine (80 mg/kg) plus xylazine (8 mg/kg), as previously described [15]. Control animals underwent sham operations.

### 4.3. Experimental Procedures

#### 4.3.1. Anxiety and Exploratory Behavior in Mice

The mice’s anxiety and exploratory behavior was evaluated by using the openfield test, the dark/light box test and the elevated maze test (Table 2). All mice were examined before the induction of CRF (*i.e.*, at 7 weeks of age; *n* = 12 for both CRF mice and non-CRF mice) and then 4 and 10 weeks after CRF induction (*i.e.*, at 14 and 20 weeks of age; *n* = 11 for both CRF mice and non-CRF mice). Experiments were performed on the same mice before CRF induction, and 4 and 10 weeks afterwards.

**Table 2:** Experimental tests and parameters evaluated.

Tests	Parameters examined
The openfield test	Anxiety, locomotor activity
The dark/light box test	Anxiety
The elevated maze test	Anxiety, time taken to make a decision
The Y maze	Recognition
The prehensile test	Post-stroke evaluation

##### 4.3.1.1. The Openfield Test [30]

Individual mice were placed inside a transparent cage (53 cm × 34.5 cm × 26 cm) for 5 min. A video system enabled us to monitor the mice throughout the experiment. Time spent in the center of the field and at the edges (in percent) and distance traveled in the center and at the edges (in cm) were recorded.

##### 4.3.1.2. The Dark/Light Box Test [31,32]

The dark light box (46 cm × 27 cm × 30 cm) is divided lengthways into two parts by a wall (46 cm × 30 cm) in which a passage enables a mouse to pass from one side to the other. One half of the box is illuminated and the other is covered and is thus in the dark. This test is based on the mouse’s aversion for light and exploratory behavior when placed in a stressful environment (such as the dark/light box). Individual mice were placed in the dark/light box for 5 minutes. Time spent in the dark part and time spent in the light part (in percent) were recorded.

##### 4.3.1.3. The Elevated Maze [33]

This test explores anxiety-related behavior and the time needed to make a decision. The maze consists of an elevated cross with four arms (25 cm × 5 cm), of which two are open and two are closed. The two closed arms are compartmentalized with 16-cm-high boards. The cross’s center (measuring 5 cm × 5 cm) is defined as the neutral zone. This test is based on the mouse’s aversion for heights and open space. The time spent in the neutral zone corresponds to the time needed to make a decision. Individual mice were placed in the maze for 5 min. Time respectively spent in the opened arms, closed arms and the neutral zone (in percent) was recorded.

#### 4.3.2. Recognition

##### The Y Maze [34]

We examined recognition in mice after 6 and 10 weeks of CRF (*i.e.*, in mice that were 16 and 20 weeks old, respectively). Hence, four groups of mice were used in this study: 16-week-old mice without CRF (the “non-CRF-6w” group) or having been exposed to 6 weeks of CRF (the “CRF-6w” group) and 20-week-old mice without CRF (the “non-CRF-10w” group) or having been exposed to 10 weeks of CRF (the “CRF-10w” group).

The mice recognition capacity was examined by using a Y maze test. The maze consists of three equally spaced arms. The mouse was placed at one end of one arm. Access to the second arm was closed and food was placed at the end of the third arm. The mouse was allowed to freely explore the two arms until it discovered the food. The experiment was then repeated with all three arms open. The test was positive if the mouse went straight to the arm with the food. Prior to the test, access to food was restricted (resulting in loss of 15% of the animal’s bodyweight) as an incitement to go directly to the food. Recognition was tested on days 1, 2, 3 and 7. Each individual mouse underwent 10 tests on each test day. The results are quoted as the percentage of positive tests per day.

#### 4.3.3. Ischemic Stroke

We induced ischemic lesions in mice after 34 weeks of CRF (*i.e.*, in 44-week-old mice). Thus, two groups of mice were used in these experiments: 44-week-old mice without CRF (the “non-CRF-34w” group) or having been exposed to 34 weeks of CRF (the “CRF-34w” group).

We used a murine model of brain ischemia-reperfusion that has been described previously [35]. In mice anesthetized with ketamine (80 mg/kg) plus xylazine (8 mg/kg), the right middle cerebral artery was occluded for 60 min with a silicone-coated filament (Doccol^®^, Sharon, Massachusetts, USA) inserted into the right common carotid artery and advanced via the internal carotid artery. The filament diameter was selected according to the mouse’s bodyweight. After an hour of ischemia reperfusion was achieved by pulling out the filament. All animals were allowed to survive for up to 3 days. Neurological evaluation was performed on days 1, 2 and 3 after the induction of stroke. 

##### 4.3.3.1. The Neuroscore

A six-grade neuroscore was used to assess post-ischemic motor and behavioral impairments [36]. Mice were graded as follows. Grade 5: mice were held gently by the tail, suspended one meter above the ground and observed for forelimb flexion. Normal mice extended both forelimbs toward the floor. Mice that extended both forelimbs toward the floor and did not display other neurological impairments were assigned a grade of 5. Grade 4: mice with consistent flexion of the forelimb contralateral to the injured hemisphere (varying from mild wrist flexion and shoulder adduction to severe posturing with full flexion of wrist and elbow, and induction of the shoulder with internal rotation) were assigned a grade of 4. Grade 3: mice were placed on a large sheet of soft, plastic-coated paper that they could grip firmly with their claws. The experimenter held the mouse by the tail and applied gentle lateral pressure at the animal’s shoulder until the forelimbs slid several centimeters. The maneuver was repeated several times to the left and to the right. Normal mice and slightly impaired mice resisted sliding to an equivalent extent in each direction. However, severely impaired mice with consistently reduced resistance to pushing towards the paretic side were assigned a grade of 3. Grade 2: mice were then allowed to move about freely and were observed for circling behavior when their tail was pulled. Mice that circled consistently towards the paretic side were assigned a grade of 2. Grade 1: mice were then allowed to move about freely and were observed for circling behavior. Mice that circled spontaneously and consistently toward the paretic side were assigned a grade 1. Grade 0: mice without any spontaneous motion were assigned a grade of 0.

##### 4.3.3.2. The Rotarod Test [36]

Motor coordination and physical resistance were evaluated by using an accelerating rotarod. Mice were placed on a rotating horizontal cylinder. The rotation speed was increased from 5 revolutions per minute to a maximum of 20 revolutions per minute over a 2-min period. The duration for which the mouse remained on the device (in seconds) was measured.

##### 4.3.3.3. The Prehensile Test [36]

A prehensile test was performed by using a horizontal stainless steel wire (length: 60 cm; diameter: 3 mm) placed 40 cm above a foam pad. The mouse’s forepaws were placed onto the wire and the animal was released. The time until the animal fell and the animal’s ability to grab the wire with a hind paw were measured. The tested animals were scored as follows: 3 points for holding onto the wire for more than 10 seconds; 2 points for holding on for between 5 and 10 seconds on the wire; 1 point for holding on for between 1 and 5 seconds; 0 point for not being able to hang on. An additional point was added if the animal managed to grab the wire with a hind paw.

##### 4.3.3.4. Histological Evaluation

Mice were sacrificed via the intraperitoneal administration of ketamine (240 mg/kg) and xylazine (24 m/kg). Brains were removed and frozen in isopentane for histological analysis. Twelve cryostat-cut coronal brain sections (thickness: 20 micrometers) were prepared at defined brain levels using the stereotaxic atlas from Paxinos and Watson [37] and then stained with cresyl violet. The total infarct volume was assessed by image analysis of the 12 coronal brain sections (using Image J software from the National Institutes of Health, USA) after digitization. Infarct volumes were corrected (to partially compensate for the effect of brain edema) by applying a mathematical model based on calculation of the volume of a truncated cone [38].

### 4.4. Statistical Analysis

All results are expressed as the mean ± standard error of the mean (SEM). Data were examined in a two-way analysis of variance (ANOVA) that took into account the animal’s status (the presence or absence of CRF, the duration of CRF and the interaction between the duration of CRF and its presence/absence). The threshold for statistical significance was set to *p* ≤ 0.05. All statistical analyses were performed using Statview software (SAS Institute Inc., Cary, NC, USA).

## 5. Conclusions

The present model appears to be a suitable model for investigating CRF-associated neurological disorders in mice. We showed that uremic toxicity in a murine model of CRF is associated with poor recognition performance and increased stroke severity but is not associated with a change in anxiety levels. The potential roles of specific uremic toxins and the underlying disease mechanisms remain to be elucidated. We are currently performing experiments on ischemic stroke after 6 weeks of CRF in mice, with a view to (i) establishing whether a shorter period of CRF has an effect on the infarct volume and (ii) identifying the disease pathways involved.
